# Effects of Long‐Term Thinning on Soil Nematode Communities in *Pinus taiwanensis* Plantations

**DOI:** 10.1002/ece3.74159

**Published:** 2026-08-07

**Authors:** Ruohan Wang, Weiya Xue, Hui Zhou, Fanglong Su, Cancan Zhao, Renhui Miao, Lei Su

**Affiliations:** ^1^ International Joint Research Laboratory for Global Change Ecology, School of Life Sciences Henan University Kaifeng Henan China

**Keywords:** community structure, ecological indices, long‐term thinning, *Pinus taiwanensis*, soil nematodes

## Abstract

Thinning, as a critical forest management practice, has multidimensional impacts on forest ecosystem environments. Soil nematode communities, which serve as sensitive bioindicators of ecosystem health, can effectively reflect the effects of different thinning intensities on forest ecosystems. Based on a long‐term thinning experiment with varying intensities in a *Pinus taiwanensis* plantation, this study examines how thinning influences the structure and function of soil nematode communities. The results showed that the total abundance of soil nematodes changed significantly across varying intensities of thinning (*p <* 0.05). Different trophic groups exhibited distinct responses: bacterivorous nematodes achieved the highest relative abundance under light thinning (10% removal), plant‐parasitic nematodes showed no significant change in relative abundance, while fungivorous and omnivorous‐predatory nematodes were significantly enriched under moderate thinning (20% removal). The synergistic changes in the latter two groups reflected trophic cascading effects. Moderate thinning significantly enhanced nematode diversity indices (Shannon‐Wiener index, evenness index), increased the proportion of K‐strategists, and strengthened the bacterial decomposition channel. These changes are attributed to moderate thinning better optimizing soil nutrient regimes and alleviating competitive exclusion through more rational allocation of food resources. Therefore, moderate thinning helps improve the structure of soil nematode communities and increases the complexity of belowground food webs. These changes further indicate improved ecosystem stability and support the healthy development of belowground ecosystems.

## Graphical abstract

Moderate thinning (20% tree removal) optimizes soil resource distribution and boosts soil nematode community diversity and stability in *Pinus taiwanensis* plantations. Fungivorous and omnivorous‐predatory nematodes are enriched under moderate thinning, indicating trophic cascading effects in soil food webs. Moderate thinning drives nematode life‐history strategies toward K‐strategists, enhancing soil ecosystem self‐regulatory capacity.

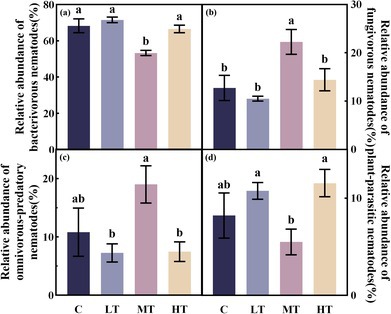

## 1 Introduction

China boasts the world's largest area of planted forest. These forests play a crucial role in soil and water conservation as well as carbon sequestration, while delivering diverse ecosystem services related to biodiversity protection, landscape preservation, and cultural education (Zhu et al. 2018). Inappropriate management practices can severely constrain the long‐term sustainability of planted forests, thereby necessitating the continuous optimization of management strategies to ensure the stability and improvement of ecosystem functions. Thinning, a key forest management intervention, involves the planned and phased selective removal of a portion of trees in immature forests based on forest growth patterns, stand structural characteristics, and predetermined management objectives (de Oliveira et al. 2021). This practice aims to create more favorable growing conditions for the remaining trees, promote stand health, and maximize the multiple benefits of forest ecosystems (Panayotov et al. 2016; Xiao et al. 2025). Appropriate thinning can effectively stimulate the growth of remaining trees, optimize stand structure, and enhance forest ecosystem stability (Kuo et al. 2023). Specifically, by reducing stand density, regulating the forest microclimate, and modifying light, temperature, and humidity conditions, thinning directly or indirectly affects soil structure and physical properties (Trentini et al. 2020). Concurrently, the natural decomposition of thinning residues can alter soil nutrient content, pH, and other chemical properties of soil (Doukalianou et al. 2022; Henneron et al. 2018). Furthermore, thinning increases understory light intensity, and the resultant improvement in light conditions influences the interactions among understory vegetation, soil microorganisms, and soil organic matter, subsequently regulating soil chemical properties (Amolikondori et al. 2020; Yu et al. 2022). These synergistic changes in soil physical and chemical properties profoundly influence soil organic matter dynamics and nutrient availability, thereby shaping habitat conditions and resource bases that structure belowground trophic interactions.

Soil nematodes account for approximately 23% of the total soil fauna and are among the most abundant groups in the soil animal community, with a broad distribution across diverse soil habitats (Anthony et al. 2023; Shao et al. 2023; Xue et al. 2026). The diversity of nematode communities is directly linked to key soil ecological functions such as soil micro‐food web regulation, organic matter decomposition and transformation, and nutrient cycling (van den Hoogen et al. 2019; Zhao et al. 2024; Gao et al. 2026). Soil nematodes directly participate in the biogeochemical cycles of key elements including carbon, nitrogen, and phosphorus, and can promptly sense and reflect subtle changes in soil health (Liu et al. 2020; Obidari et al. 2024). Additionally, nematodes exhibit traits such as high species richness, well‐defined functional trophic groups, and sensitivity to environmental changes, making them ideal bioindicators for assessing soil ecosystem health (Bongers and Ferris 1999; Gao et al. 2021; Sun et al. 2024; Ahmed et al. 2026). Therefore, analyzing the taxonomic composition, abundance, and trophic structure of nematodes can effectively characterize soil environmental quality and belowground biodiversity, providing a scientific basis for soil health evaluation and sustainable management of plantation forest ecosystems.

Sustainable management of China's planted forests relies on a clear understanding of belowground ecological processes. Nevertheless, the long‐term impacts of thinning on such processes remain poorly understood. The Dabie Mountain region lies within a climatic transition zone, where the growth of plantation forests is highly sensitive to climate change. As a commonly cultivated tree species in this region, *Pinus taiwanensis* not only exhibits strong ecological adaptability but also holds considerable economic value. However, in‐depth research on the long‐term impact mechanisms of thinning treatments with varying intensities on the structure of soil nematode communities remains scarce. To address this gap, the present study employs a long‐term thinning experimental platform in *Pinus taiwanensis* plantations. By determining changes in soil nematode communities under different thinning intensities and synchronously monitoring soil physicochemical properties, this research aims to clarify the pathways and key drivers through which thinning disturbance regulates soil nematode communities. The findings are expected to provide a theoretical basis for plantation forests management, soil biodiversity conservation, and sustainable forest ecosystems management, thereby contributing to the maintenance of ecological balance and the improvement of forest quality.

## 2 Materials and Methods

### 2.1 Study Site

The experimental study site is situated at the Huangbaishan site of the Henan Dabieshan National Field Observation and Research Station of Forest Ecosystem (31°23′–31°35′ N, 115°13′–115°20′ E, a.s.l. 400–900 m). This region lies in a transitional zone between the northern subtropical and warm temperate zone, characterized by a warm temperate monsoon climate with the features of high temperatures and abundant rainfall in summer, cold and dry winters, and synchronized heat and precipitation seasons. The mean annual temperature in this area is approximately 14.8°C, and the mean annual precipitation is about 1298.2 mm. The *Pinus taiwanensis* plantation was established in the 1960s and was 60 years old at the time of sampling. The dominant understory vegetation in the *Pinus taiwanensis* forest includes *Dryopteris chinensis*, *Lindera glauca*, *Rubus corchorifolius*, and other associated species. The primary soil types are Cambisols and Luvisols under the WRB classification system (IUSS Working Group WRB 2022).

### 2.2 Experimental Design

**FIGURE 1:**
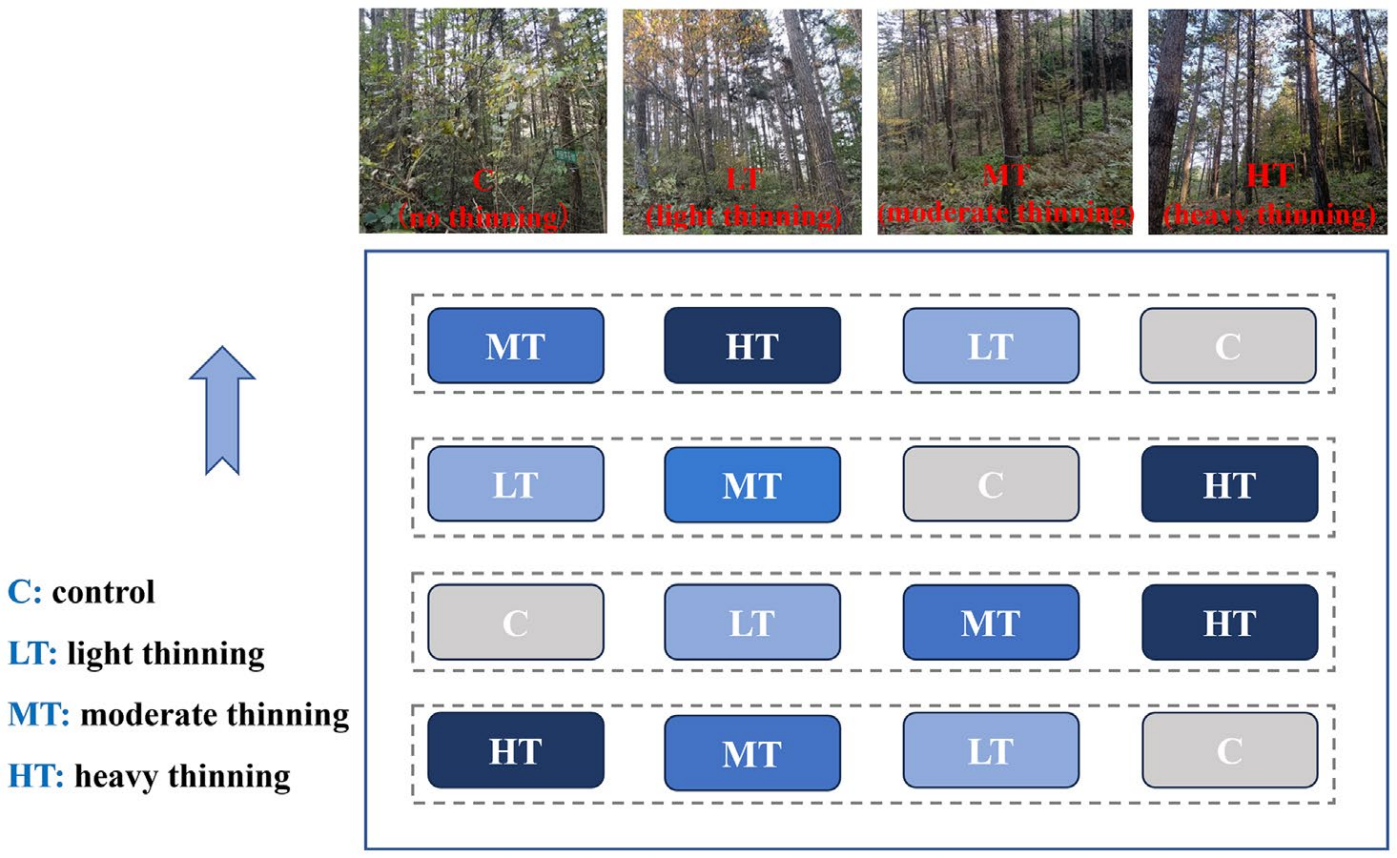
Schematic diagram of the long‐term thinning experimental platform for *Pinus taiwanensis* plantation.

The experimental *Pinus taiwanensis* plantation was established in 1964. The first thinning was conducted in 1978 when the stand was 14 years old (young forest stage). Subsequent thinnings were implemented at the middle‐aged stage (stand age 21 years, 1985), near‐mature stage (stand age 31 years, 1995), mature stage (stand age 43 years, 2007), and over‐mature stage (stand age 55 years, 2019), corresponding to the second, third, fourth, and fifth thinning events, respectively. Each thinning operation was implemented based on the retained stand from the preceding thinning event. Four thinning intensity treatments were applied for each thinning event: control (no thinning), light thinning (10% tree removal), moderate thinning (20% tree removal), and heavy thinning (30% tree removal). Thinning followed the principle of “removing dense trees to maintain uniform stand spacing, eliminating inferior trees to retain superior individuals, and considering inter‐tree distance as a supplementary criterion.” Thinning intensity was quantified and controlled according to the number of retained trees per unit area. The experiment adopted a randomized complete block design with four blocks, resulting in a total of 16 plots (Figure 1), each with dimensions of 20 m × 30 m. Blocks were spaced 10–20 m apart, and plots within each block were separated by 8–10 m buffers.

### 2.3 Soil Sampling and Nematode Extraction

Soil sampling for nematodes was conducted in August 2024. In each plot, more than ten soil cores (0–10 cm depth) were randomly collected using a 5‐cm diameter auger and thoroughly mixed into one composite sample per plot. The composite sample was then divided into subsamples for the determination of multiple soil properties, with a subsample of soil volume equivalent to three cores reserved specifically for nematode extraction. In total, 16 composite soil samples were obtained (4 thinning treatments × 4 replicate blocks). The composite samples were further homogenized and sieved through a 2 mm sieve to remove plant roots and coarse debris. The processed soil samples were then split into two parts: one was refrigerated at 4°C for subsequent nematode extraction and identification, and the other was frozen at −20°C for soil element content determination, ensuring the comprehensiveness and scientific rigor of the experimental data.

Soil nematodes were extracted via a modified Baermann funnel method (Martin 1962). A 100 g subsample of soil was wrapped in non‐woven fabric and placed in a 20 cm‐diameter funnel pre‐filled with water to eliminate air bubbles in the rubber tube. Additional water was added to submerge the soil sample, and the setup was left undisturbed for 48 h with water replenished as required. Leveraging the hydrotactic behavior and gravity of nematodes, the organisms migrated through the non‐woven fabric into the rubber tube. The clamp on the rubber tube was then opened to allow the nematode suspension to flow slowly into a pre‐prepared centrifuge tube. Approximately 10 mL of the nematode suspension was collected and centrifuged at 2500 r/min for 5 min. After discarding 5 mL of the supernatant, the remaining suspension was incubated in a 65°C constant‐temperature water bath for 3 min, followed by the addition of 5 mL of TAF fixative (water: 40% formaldehyde: triethanolamine = 91:7:2, v/v/v). The mixture was thoroughly agitated and stored at 4°C for preservation.

### 2.4 Determination of Soil Nematode Community Indices

The abundance of soil nematodes was determined by counting all individuals in a fixed volume of the extracted suspension using a counting chamber under an optical microscope. The number of soil nematodes per 100 g of dry soil was calculated based on the sample count, the fresh soil mass used for extraction, and the corresponding soil moisture content.

Nematodes were further identified to the genus level based on morphological characteristics (Yin 1998) for taxonomic composition analysis. The procedure for analyzing the trophic group composition and group‐specific abundance of soil nematodes was as follows: 100 nematodes were randomly selected from each sample for observation if more than 100 individuals occurred in the sample; otherwise, all individuals were examined under an optical microscope. Based on established feeding habit and habit classifications, nematode genera were categorized into four trophic groups: Plant‐Parasitic Nematodes (Pp), Fungivorous Nematodes (Fu), Bacterivorous Nematodes (Ba), and Omnivorous‐Predatory Nematodes (Op) (Bongers 1990; Yeates et al. 1993). The number of individuals in each group was counted, and their abundances were calculated using the same method as for total soil nematode community abundance.

Nematode community indices were computed based on the life‐history strategies of soil nematodes. First, nematodes were classified into five colonizer‐persister (cp) groups (cp1–cp5). Combined with trophic group and abundance data, the following indices were calculated: soil nematode genus richness (GR), Nematode Channel Ratio (NCR), predator‐to‐herbivore nematode abundance ratio (RF), Shannon‐Wiener diversity index (*H*′), evenness index (*J*′), Simpson's dominance index (*λ*), Maturity Index (MI), Plant Parasitic Index (PPI), Basal Index (BI), Enrichment Index (EI), Structure Index (SI), and Channel Index (CI) (Bongers and Bongers 1998; Zhao et al. 2024).

### 2.5 Determination of Soil Physicochemical Properties

Sealed soil samples were retrieved from the refrigerator, sieved through a 1 mm mesh, and air‐dried before weighing 10 g into a beaker. Subsequently, 25 mL of purified water was added, followed by stirring and standing for 30 min. Soil pH was measured using a Sartorius PB‐10 pH meter. During soil sampling, soil temperature (ST) was quantified using a STEPS field soil temperature sensor. Soil water content (SWC) was determined by oven‐drying 20 g of fresh soil at 105°C for 48 h.

Soil nutrient contents were determined as follows: total phosphorus (TP) was analyzed via the H_2_SO_4_‐HClO_4_ digestion method combined with the molybdenum‐antimony colorimetric method; total nitrogen (TN) was quantified using the Kjeldahl method; total carbon (TC) and total organic carbon (TOC) were both determined by the potassium dichromate oxidation‐external heating method; nitrate nitrogen (NO_3_^−^‐N) was measured using ultraviolet spectrophotometry; ammonium nitrogen (NH_4_^+^‐N) was analyzed via the indophenol blue colorimetric method. Particulate organic carbon (POC) and mineral‐associated organic carbon (MAOC) were separated by wet sieving and quantified using the potassium dichromate oxidation method.

### 2.6 Statistical Analysis

Prior to conducting statistical analyses, all data were tested for normality and homogeneity of variance. The effects of different thinning intensities on soil physicochemical properties and nematode communities were assessed using one‐way analysis of variance (ANOVA). If the data met the assumptions of normality and homogeneity of variance following transformation, multiple comparisons were performed using the least significant difference (LSD) method. All the aforementioned statistical analyses were conducted using SPSS 27.0 software, with the significance level set at *p* = 0.05, and correlation analyses between nematode communities and soil physicochemical properties were also implemented in this software. Furthermore, two‐dimensional faunal analysis, based on the Structure Index (SI) and Enrichment Index (EI), and permutational multivariate analysis of variance (PERMANOVA) to test differences in soil nematode community structure among thinning treatments, were performed using the dplyr, ggplot2 and vegan packages in R software (version 4.2.2) to quantify the resource allocation patterns and stability characteristics of the soil food web. Principal Coordinates Analysis (PCoA) was carried out using Canoco 5 software to reveal differences in soil nematode community structure among different thinning treatment groups.

## 3 Results

### 3.1 Soil Physicochemical Properties

As shown in Table 1, significant differences were detected in soil total phosphorus (TP) among different thinning treatments (*F* = 9.460, *p* = 0.002), with the control and light thinning groups exhibiting significantly higher TP contents than the moderate and heavy thinning groups (*p* < 0.001). Soil water content (SWC) differed extremely significantly across thinning treatments (*F* = 12.648, *p* < 0.001), where the heavy thinning group had a significantly higher SWC than the other treatments (*p* < 0.001), and both the light and heavy thinning groups showed significantly higher SWC than the control group (*p* < 0.05). Total nitrogen (TN) content in the light and heavy thinning groups was significantly greater than that in the control and moderate thinning groups (*p* < 0.05). No significant differences were observed among the different thinning treatments for the other soil parameters (*p* > 0.05).

**TABLE 1:** Soil physical and chemical properties under different thinning treatments (mean ± SE).

Treatment	C	LT	MT	HT
SWC (%)	28.74 ± 1.59c	36.56 ± 2.14b	35.95 ± 3.33b	47.71 ± 1.09a
ST (°C)	25.88 ± 1.45a	30.47 ± 2.25a	26.56 ± 1.51a	29.33 ± 2.41a
pH	5.11 ± 0.14a	5.02 ± 0.12a	5.25 ± 0.09a	5.28 ± 0.08a
TP (g/kg)	0.58 ± 0.02a	0.63 ± 0.05a	0.41 ± 0.03b	0.48 ± 0.03b
TN (g/kg)	2.76 ± 0.16b	3.91 ± 0.48a	3.01 ± 0.27ab	3.88 ± 0.36a
TC (g/kg)	37.54 ± 2.65a	46.31 ± 6.10a	37.30 ± 3.85a	46.37 ± 3.53a
NO_3_^−^‐N (g/kg)	0.45 ± 0.03ab	0.56 ± 0.01a	0.37 ± 0.03b	0.33 ± 0.08b
NH_4_^+^‐N (g/kg)	4.80 ± 0.22ab	4.98 ± 0.40ab	4.04 ± 0.26b	5.33 ± 0.34a
TOC (g/kg)	38.96 ± 4.41a	41.07 ± 4.42a	44.34 ± 5.28a	42.09 ± 0.32a
POC (g/kg)	13.64 ± 3.79a	15.07 ± 2.93a	15.44 ± 3.75a	14.34 ± 2.91a
MAOC (g/kg)	25.32 ± 0.89a	26.00 ± 3.07a	28.91 ± 2.39a	27.75 ± 2.60a

*Note:* Different letters indicate significant differences among treatments (*p* < 0.05).

Abbreviations: C, control; HT, heavy thinning; LT, light thinning; MAOC, soil mineral‐associated organic carbon; MT, moderate thinning; NH_4_^+^‐N, soil ammonium nitrogen; NO_3_^−^‐N, soil nitrate nitrogen; POC, soil particulate organic carbon; ST, soil temperature; SWC, soil water content; TC, soil total carbon content; TN, soil total nitrogen content; TOC, soil total organic carbon; TP, soil total phosphorus.

### 3.2 Abundance of Soil Nematode Communities

**FIGURE 2:**
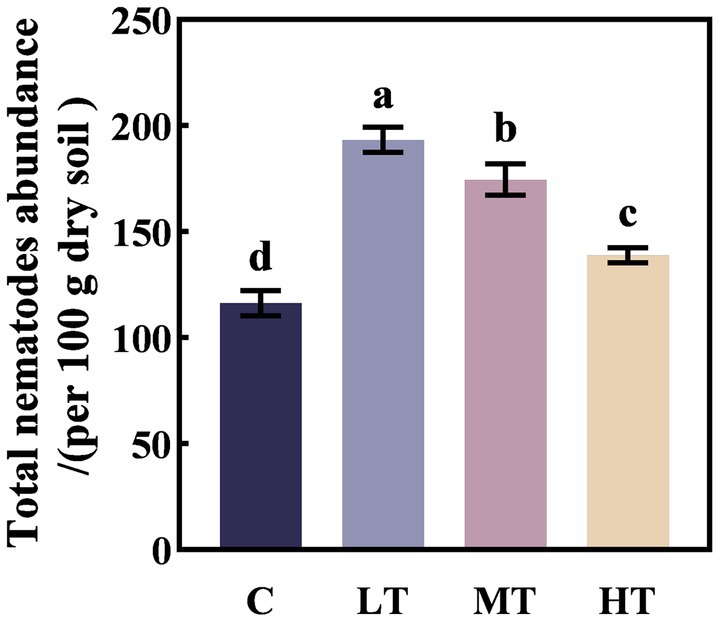
Total abundance of soil nematode communities under different thinning treatments (mean ± SE). See Table 1 for abbreviation.

Thinning intensities had a significant effect on the total abundance of soil nematode communities (*F* = 34.948, *p* < 0.001). Relative to the control group, the light thinning, moderate thinning, and heavy thinning treatments increased the total abundance by 66.28% (*p* < 0.001), 50.16% (*p* < 0.001), and 19.40% (*p* = 0.019), respectively. Overall, the total abundance of soil nematodes exhibited a unimodal response to thinning intensities, increasing initially and then declining, with the peak total abundance observed in the light thinning treatment (Figure 2).

### 3.3 Soil Nematode Trophic Group

**FIGURE 3:**
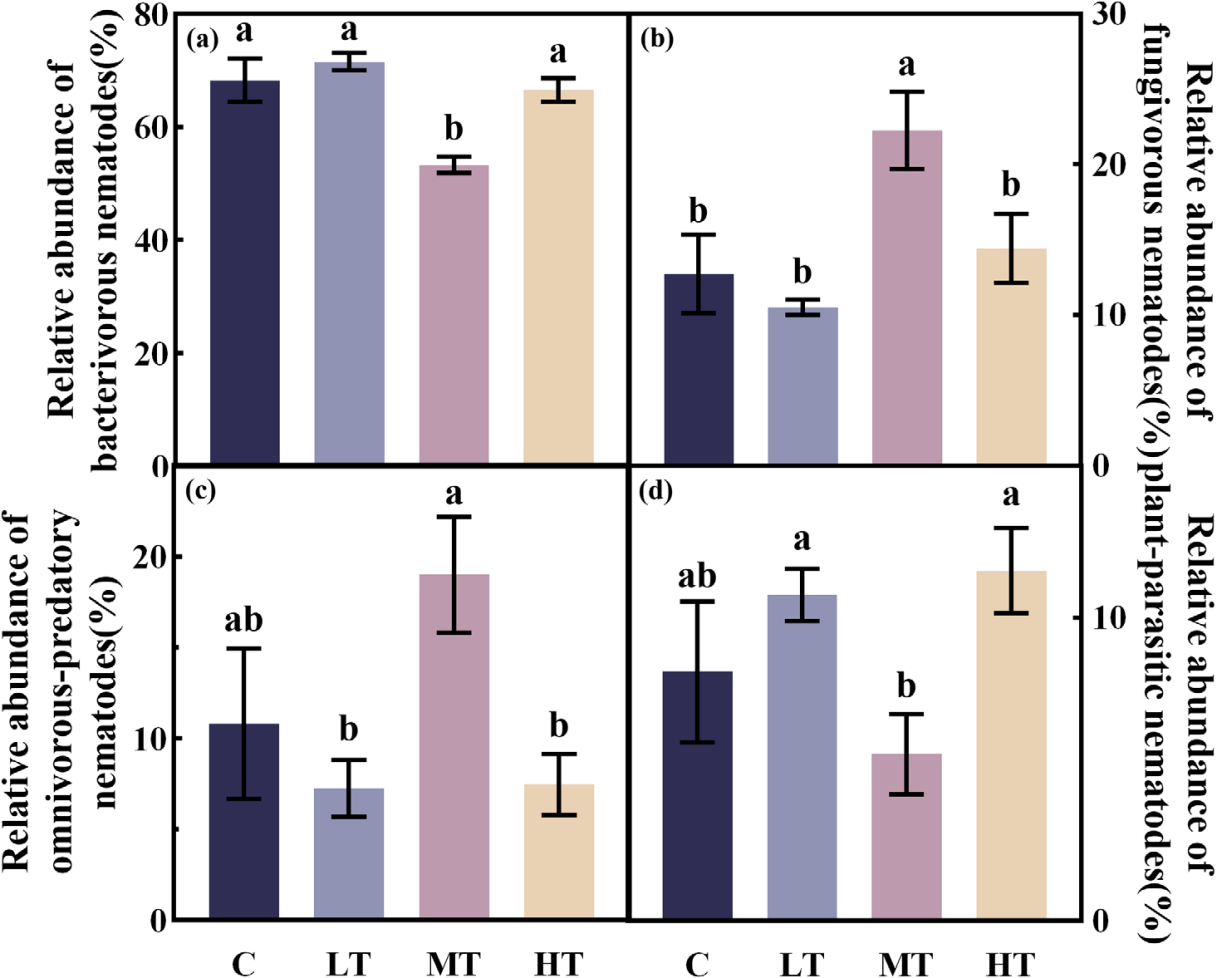
Relative abundance of different trophic groups in soil nematode communities under different thinning treatments (mean ± SE). (a) Relative abundance of bacterivorous nematodes; (b) Relative abundance of fungivorous nematodes; (c) Relative abundance of omnivorous‐predatory nematodes; (d) Relative abundance of plant‐parasitic nematodes. See Table 1 for abbreviation.

The results also demonstrated that different thinning treatments exerted variable effects on the trophic groups of the soil nematode community. A significant difference was detected in the relative abundance of bacterivorous nematodes (Ba) across different thinning treatments (*F* = 11.154, *p* < 0.001; Figure 3a). The moderate thinning treatment significantly reduced the relative abundance of Ba compared to the control, light thinning, and heavy thinning treatments by 28.13% (*p* < 0.001), 34.27% (*p* < 0.001), and 19.98% (*p* = 0.002), respectively. Similarly, the relative abundance of fungivorous nematodes (Fu) responded significantly among treatments (*F* = 5.549, *p* = 0.013; Figure 3b). In contrast to this, the relative abundance of Fu under the moderate thinning treatment was significantly higher than that under the control, light thinning, and heavy thinning treatments, with increases of 42.73% (*p* = 0.009), 52.80% (*p* = 0.002), and 35.17% (*p* = 0.025), respectively. The relative abundance of omnivorous‐predatory nematodes (Op) also differed significantly among treatments (*F* = 3.719, *p* = 0.042; Figure 3c). The moderate thinning treatment supported a significantly higher relative abundance of Op compared to the light and heavy thinning treatments, which were lower by 61.84% (*p* = 0.013) and 60.64% (*p* = 0.014), respectively. Conversely, the relative abundance of plant‐parasitic nematodes (Pp) was significantly higher in the light and heavy thinning treatments than in the moderate thinning treatment by 95.45% (*p* = 0.036) and 109.99% (*p* = 0.019), respectively (Figure 3d).

### 3.4 Soil Nematode Ecological Indices and Life‐History Groups

**FIGURE 4:**
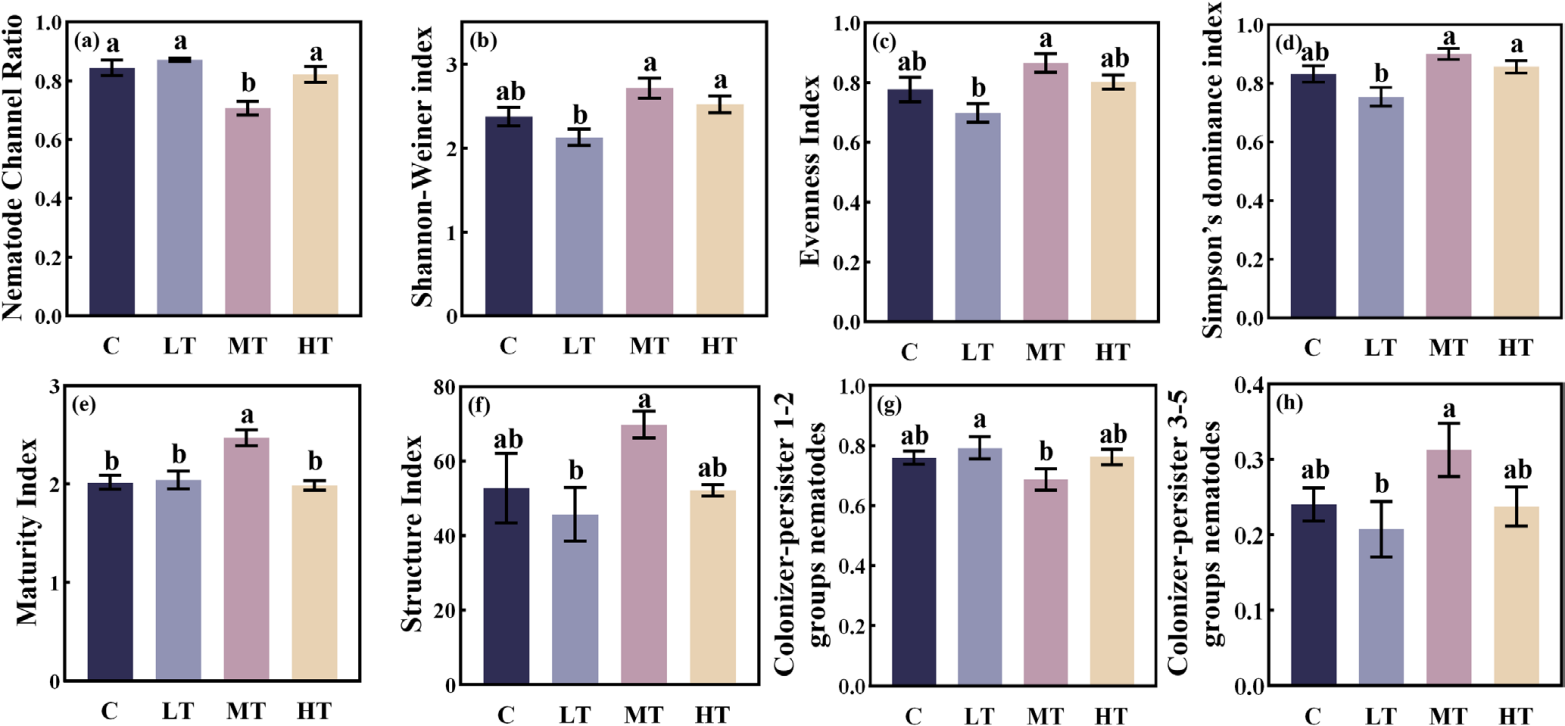
Ecological indices of soil nematode communities under different thinning treatments (mean ± SE). (a) Nematode Channel Ratio; (b) Shannon‐Weiner index; (c) Evenness index; (d) Simpson's dominance index; (e) Maturity Index; (f) Structure Index; (g) Colonizer‐persister 1‐2 groups nematodes; (h) Colonizer‐persister 3‐5 groups nematodes. See Table 1 for abbreviation.

The functional indices of the soil nematode community responded variably to different thinning intensities. A highly significant effect was found for the nematode channel ratio (NCR) among different thinning treatments (*F* = 10.544, *p* = 0.001; Figure 4a). The NCR under the moderate thinning group was significantly higher than the control, light thinning, and heavy thinning treatments by 19.32% (*p* < 0.001), 23.22% (*p* < 0.001), and 16.22% (*p* = 0.003), respectively. Significant differences were observed in the Shannon‐Wiener diversity index (*H′*) of the soil nematode community across the thinning treatments (*F* = 5.284, *p* = 0.015; Figure 4b). The moderate thinning group resulted in a significantly higher *H′* compared to the light thinning (27.50% increase, *p* = 0.002) and control (14.18% increase, *p* = 0.046) treatments. The heavy thinning group also showed a higher *H′* than the light thinning treatment (18.39% increase, *p* = 0.024). Significant differences in the evenness index (*J'*) of the soil nematode community were found among the treatments (*F* = 4.479, *p* = 0.025; Figure 4c), with the moderate thinning group significantly increasing by 23.93% (*p* = 0.004) and the heavy thinning group by 14.74% (*p* = 0.046) relative to the light thinning group.

The Simpson diversity index (*λ*) was also affected (*F* = 5.857, *p* = 0.011; Figure 4d), being significantly higher in the moderate (19.44% increase, *p* = 0.002) and heavy (13.60% increase, *p* = 0.024) thinning treatments relative to the light thinning treatment. The maturity index (MI) showed a significant difference (*F* = 9.669, *p* = 0.002; Figure 4e). The MI under moderate thinning was significantly higher than in the control (22.57%, *p* < 0.001), light thinning (21.05%, *p* = 0.001), and heavy thinning (24.38%, *p* < 0.001) treatments. Additionally, the structure index (SI) of the moderate thinning group was significantly 52.40% higher compared to the light thinning treatment (*p* = 0.018; Figure 4f). Other soil nematode community indices also displayed significant differences.

The results further revealed differential effects of thinning on nematodes' life‐history strategies, based on colonizer‐persister (cp) classes. The relative abundance of cp 1–2 nematodes was significantly 15.27% higher in the light thinning treatment than in the heavy thinning treatment (*p* = 0.032; Figure 4g). Conversely, the relative abundance of cp 3–5 nematodes was 50.60% higher in the moderate thinning group compared to the light thinning treatment (*p* = 0.032; Figure 4h).

**FIGURE 5:**
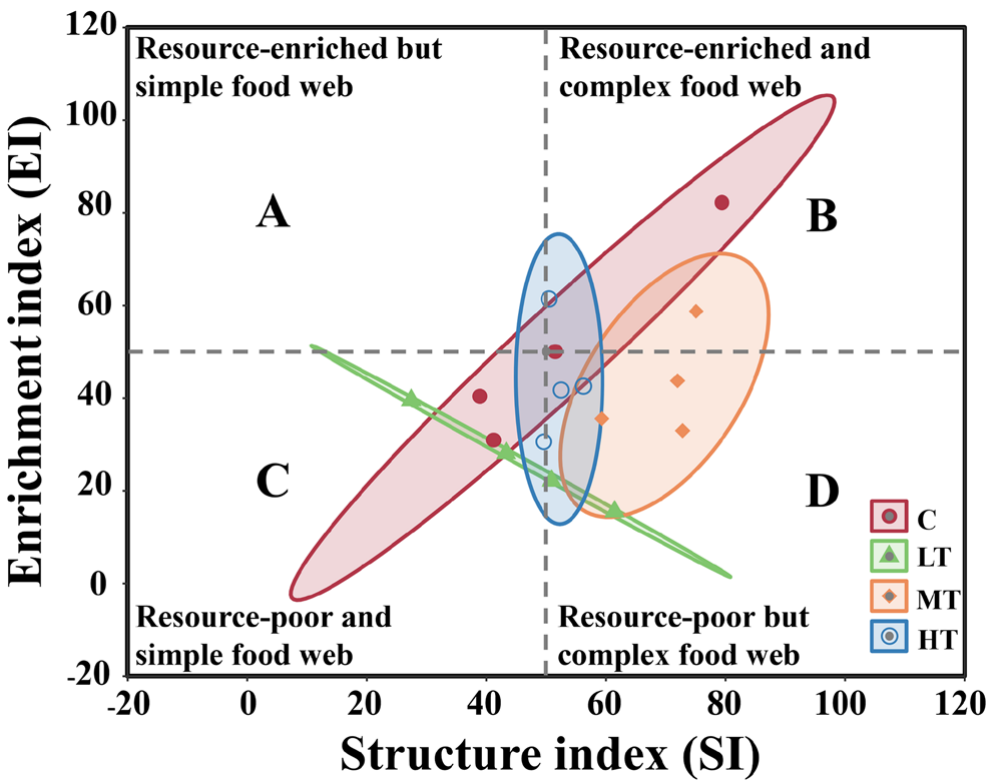
Trophic group analysis of soil nematode communities under different thinning treatments.

In parallel, the functional status of the soil food web across different treatments was evaluated using a two‐dimensional faunal analysis plot based on the structure index (SI) and enrichment index (EI) to characterize the trophic structure of nematode communities (Figure 5). The plot revealed distinct spatial segregation among sample points from different treatments: samples from the control treatment were primarily distributed in quadrants B and C, with relatively wide confidence ellipses, indicating high soil resource enrichment and complex food web structure in this treatment; samples from the moderate thinning treatment were mainly located in quadrants B and D, suggesting relatively lower resource enrichment but sustained food web complexity; and sample points from the light and heavy thinning treatments were positioned between the control and moderate thinning treatments, with overlapping confidence ellipses. The degree of inter‐group overlap indicates potential cross‐over effects of the treatments on soil food web regulation, while the separation of confidence ellipses reflects the unique effects of each treatment.

### 3.5 Relationships Between Soil Physicochemical Properties and Soil Nematode Communities

**FIGURE 6:**
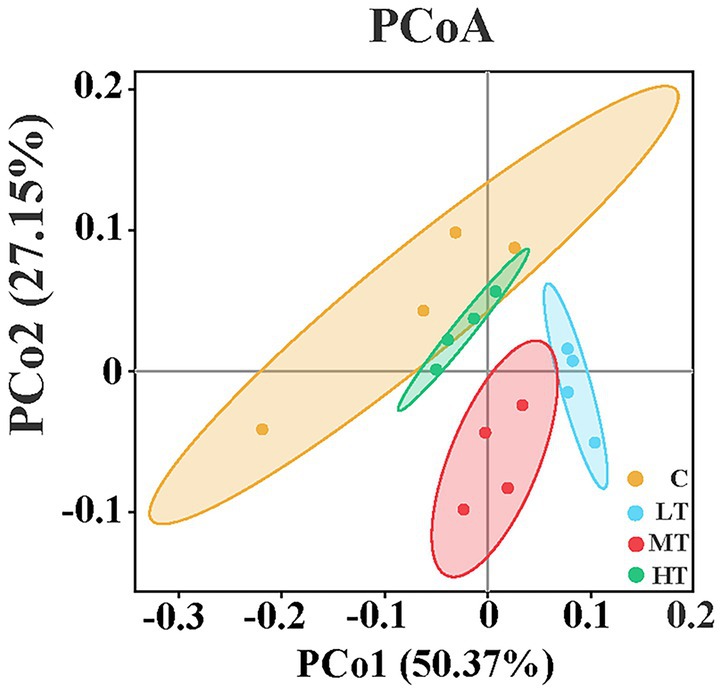
Principal Coordinate Analysis (PCoA) of soil nematode community structure under different thinning intensities.

Principal Coordinate Analysis (PCoA) showed that PCo1 and PCo2 collectively explain 77.52% of the total variation, effectively capturing the core differences among samples (Figure 6). In terms of distribution patterns, samples from the control treatment clustered in the negative region of PCo1, with relatively broad confidence ellipses, indicating higher within‐group variability in community characteristics under this treatment. In contrast, samples from the moderate thinning treatment aggregated in the positive region of PCo1, displaying independent and compact confidence ellipses, which suggested strong within‐group consistency in community structure. A clear separation trend was evident between the moderate thinning and control treatments, reflecting a distinct regulatory effect of moderate thinning on the community compared to the control. Samples from the light thinning treatment clustered near the moderate thinning treatment, with partial overlapping confidence ellipses. Samples from the heavy thinning treatment were situated in a transitional zone between the control and moderate thinning treatments, with confidence ellipses overlapping those of the control group. PERMANOVA indicated that thinning treatment explained 26.6% of the variation in community structure (*R* ^2^ = 0.266), and separation among treatments did not reach statistical significance (*p* = 0.072), though directional separation was visible in the ordination. Light and heavy thinning treatments occurred in transitional zones between the control and moderate thinning treatments, consistent with intermediate community characteristics. This spatial pattern aligned with the weak trend detected by PERMANOVA, suggesting a potential gradient in nematode community structure along the thinning intensity gradient; the moderate thinning treatment was positioned furthest from the control in the ordination space.

**FIGURE 7:**
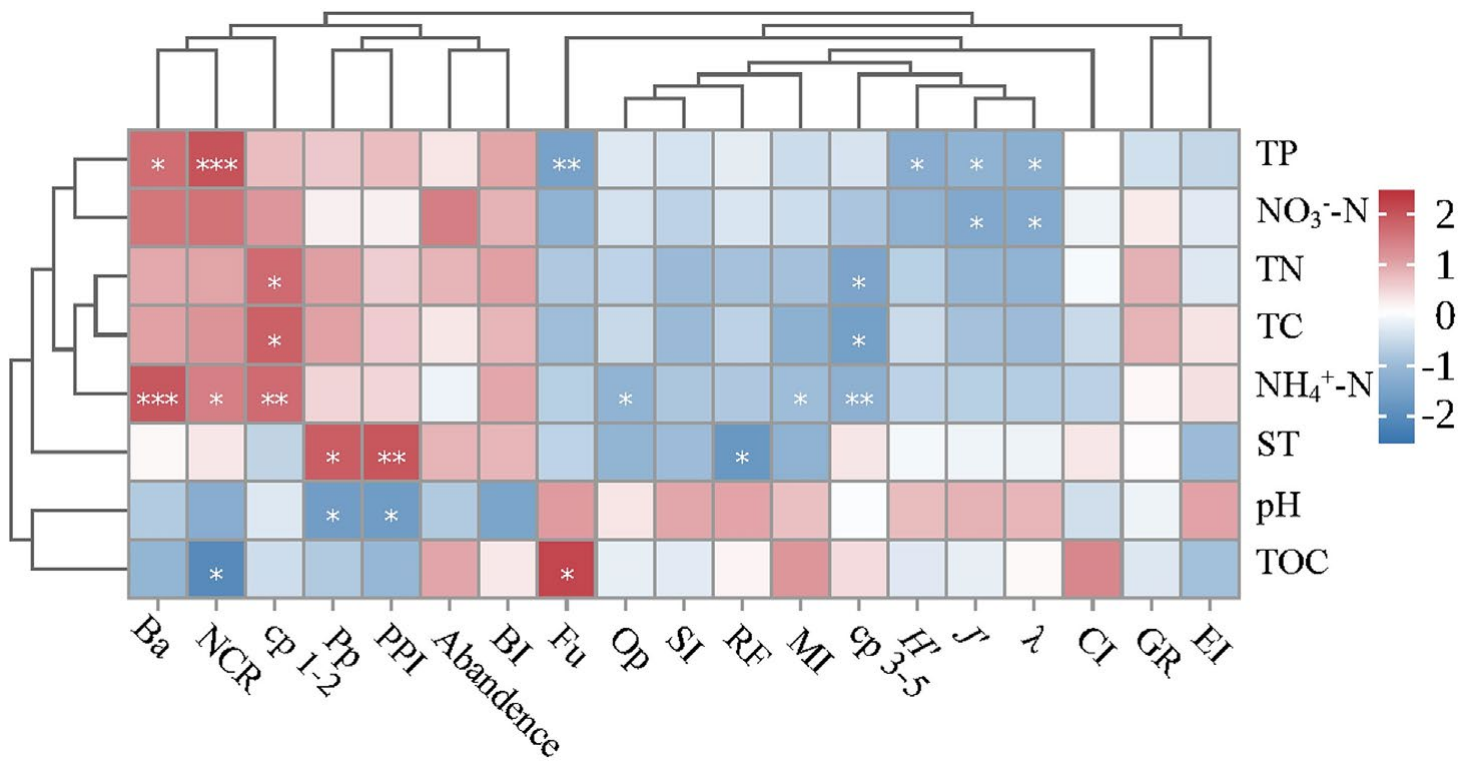
Correlation analysis between soil nematode community composition, ecological indices and environmental factors (*p < 0.05, **p < 0.01, ***p < 0.001). *λ*, Simpson's dominance index; Abandence, total abundance of soil nematode communities; Ba, bacterivorous nematodes; BI, basal index; CI, channel index; cp 1–2, colonizer‐persister 1–2; cp 3–5, colonizer‐persister 3–5; EI, enrichment index; Fu, fungivorous nematodes; GR, genus richness; *H′*, Shannon‐Weiner index; *J'*, evenness index; MI, maturity index; NCR, nematode channel ratio; Op, omnivorous‐predatory nematodes; Pp, plant‐parasitic nematodes; PPI, plant‐parasitic nematode index; RF, predator‐to‐herbivore nematode abundance ratio; SI, structure index. Abbreviations of soil physicochemical factors are presented in Table 1.

Correlation heatmap and cluster analysis were employed to explore the relationships between soil physicochemical properties and soil nematode community structure (Figure 7). The results revealed distinct clustering patterns between environmental factors and nematode community indices: within the environmental factor cluster, TP, TN, total carbon (TC), nitrate nitrogen (NO_3_^−^‐N), and ammonium nitrogen (NH_4_^+^‐N) grouped together, while soil temperature (ST), pH, and total organic carbon (TOC) formed a separate cluster. Among nematode community indices, Ba, NCR, cp 1–2, plant parasitic index (PPI), total abundance, and BI clustered into one major group, with the remaining indices forming another major group. This pattern suggests that different categories of environmental factors exert more specific influences on distinct sets of nematode indices.

Specifically, TP exhibited a significant positive correlation with Ba and NCR, and a significant negative correlation with Fu, *H′*, *J′*, and *λ*; NO_3_^−^‐N was significantly negatively correlated with *J'* and *λ*; TN, TC, and NH_4_^+^‐N were all significantly positively correlated with cp 1–2 and significantly negatively correlated with cp 3–5; NH_4_^+^‐N was significantly positively correlated with Ba and NCR, and significantly negatively correlated with Op and MI; Pp and PPI were significantly positively correlated with ST and significantly negatively correlated with pH; ST was significantly negatively correlated with predator‐to‐herbivore nematode abundance ratio (RF); TOC was significantly negatively correlated with NCR and significantly positively correlated with Fu. Notably, the cluster tree analysis results corresponded to some extent with the strength of correlations: environmental factors within the same cluster displayed more consistent directions and magnitudes of influence on nematode indices belonging to the same category.

## 4 Discussion

### 4.1 Effects of Different Thinning Treatments on the Total Abundance and Trophic Group Composition

The present study revealed that the total abundance of soil nematode communities followed a nonlinear trend with increasing thinning intensity, initially increasing and then declining. The highest total abundance was recorded in the light thinning treatment, while the lowest was in the control. This result is consistent with the findings of Yang et al. (2018), who reported that moderate forest management practices enhance nematode abundance. The underlying mechanism is likely that thinning reduces stand density, thereby improving understory light availability and ventilation conditions, which fosters more vigorous growth of understory vegetation (Zhao, Guo, et al. 2021). This, in turn, provides more favorable habitat and nutritional resources for nematodes, significantly promoting the total abundance of the community. However, as thinning intensity increases further, reduced canopy cover may intensify soil moisture evaporation, which could inhibit nematode reproduction and lead to a decline in total abundance. This pattern supports the “intermediate disturbance hypothesis”, which posits that moderate thinning promotes soil biodiversity (Kuo et al. 2023).

Different thinning treatments exerted distinct effects on various nematode trophic groups, primarily due to the directional regulation of soil physicochemical properties on food resources distribution. Both the relative abundance of bacterivorous nematodes (Ba) and the total nematode abundance peaked in the light thinning treatment. Notably, total phosphorus (TP) content exhibited a significant positive correlation with the abundance of Ba—a phenomenon also observed by Furmanczyk et al. (2025). Soil phosphorus content did not increase continuously with thinning intensity, though moderate thinning has been shown to significantly enhance soil phosphorus levels (Tian et al. 2019; Yang et al. 2024), thereby promoting the relative abundance of Ba. Inadequate phosphorus supply can constrain the growth of bacterial communities (Oliverio et al. 2020), reducing food availability for Ba and consequently lowering their relative abundance.

The relative abundances of fungivorous nematodes (Fu) and omnivorous‐predatory nematodes (Op) were both highest in the moderate thinning group, and their trends were consistent. This coordinated change conforms to the patterns of trophic cascades in soil food webs (Pires et al. 2023). Fu primarily feed on soil fungi, whose growth and reproduction depend on the supply of soil organic carbon (Jörgensen et al. 2021; Kitagami and Makita 2025). Thinning promotes understory vegetation and fine root growth, increases organic matter input, and enhances aggregate‐associated carbon storage (Ma et al. 2018), thereby significantly increasing soil organic carbon reserves in plantations (Gong et al. 2021). The present study showed that both total organic carbon (TOC) and mineral‐associated organic carbon (MAOC) contents were highest under moderate thinning. The abundant organic carbon provided sufficient carbon sources for fungi, facilitating Fu proliferation. As higher trophic groups, Op rely on the accumulation of lower trophic‐level nematodes for resource supply, and the enrichment of Fu provides them with stable prey resources (Maina et al. 2021).

No significant differences in the relative abundance of plant‐parasitic nematodes (Pp) were detected among treatments, which may be attributed to their distribution being closely tied to root characteristics, soil pH, temperature, and other factors (Jakubcsiková et al. 2023). In this study, the 60‐year‐old *Pinus taiwanensis* plantation had a mature root system, making it difficult for thinning to significantly alter the types and quantities of root exudates. Additionally, soil pH showed no significant variations, and temperature fluctuations did not exceed the adaptive thresholds of Pp, thus maintaining their relative abundance stability.

### 4.2 Effects of Different Thinning Treatments on Soil Nematode Community Ecological Indices

The present study revealed that the Shannon‐Wiener diversity index (*H′*), evenness index (*J'*), and Simpson's dominance index (*λ*) in the moderate thinning treatment were significantly higher than those in other treatments, indicating that the species richness and distribution evenness of the nematode community reached an optimal state under moderate thinning. This supports the existing view that “moderate disturbance can enhance nematode community diversity” (Pan et al. 2020; Zhao, Chen, et al. 2021). Regarding soil physicochemical properties, although TP was lower in the moderate thinning plots, the levels of total nitrogen (TN), total carbon (TC), and TOC were moderately high. This balanced nutrient distribution alleviated competitive exclusion within a single trophic group, thereby providing ecological niches for nematode taxa with distinct ecological requirements. Simultaneously, the soil water content (SWC) in this treatment was moderate, avoiding the reduced nematode reproduction associated with prolonged low humidity in the control (Bristol et al. 2023) and mitigating the potential adverse effects of high humidity on certain nematode taxa under heavy thinning, thereby further enhancing community diversity and evenness. In contrast, diversity indices were lower in the light thinning treatment, which may be associated with the excessive dominance of Ba in this treatment. Lazarova et al. (2021) similarly reported that excessive dominance by a single trophic group can reduce community diversity.

Changes in the maturity index (MI) and structure index (SI) reflect the regulatory effect of thinning on the stability of the soil ecosystem (Du Preez et al. 2022; Ney et al. 2019). The MI in the moderate thinning treatment was significantly higher than in other treatments (*p* < 0.001), and the SI was also significantly higher compared to the light thinning treatment, indicating greater soil ecosystem stability and a more complex food web structure under moderate thinning. This result complements the two‐dimensional faunal analysis, where moderate thinning samples were mainly distributed in quadrant D, characterized by “limited resources but complex food webs,” reflecting that the nematode community in this treatment enhanced ecosystem resistance to disturbance by optimizing its trophic structure. Mechanistically, the relative abundance of colonizer‐persister (cp) 3–5 groups (i.e., K‐strategist nematodes) was significantly higher under moderate than under light thinning. K‐strategist nematodes require higher environmental stability, and their enrichment contributes to improving the community's self‐regulatory capacity (Bongiorno et al. 2019). Correlation analysis showed that MI was significantly positively correlated with Op and negatively with Ba. Under moderate thinning, the relative abundance of Op was the highest, while that of Ba was the lowest, further indicating that the richness of higher trophic groups can promote community maturity.

The nematode channel ratio (NCR) was significantly highest under moderate thinning, reflecting the regulatory effect of thinning on the decomposition pathways of soil organic matter. A higher NCR value indicates greater dominance of the bacterial decomposition channel (Mulder et al. 2005). The elevated NCR under moderate thinning may stem from the synergistic interaction between bacterial and fungal decomposition pathways: the relatively lower TP may constrain bacterial decomposition efficiency, while higher TOC promotes fungal decomposition (Tian et al. 2024). This synergy not only enhances organic matter decomposition efficiency but also provides diverse food resources for different nematodes trophic groups. Furthermore, correlation analysis revealed a significant negative correlation between TOC and NCR, as well as a significant positive correlation between TOC and Fu, further confirming the regulatory role of the enhanced fungal decomposition pathway under moderate thinning in shaping NCR.

Changes in cp groups reveal how thinning regulates the life history strategies of soil nematodes. The results showed that the relative abundance of cp 1–2 groups (r‐strategist nematodes) was significantly higher under light thinning than under moderate thinning, while the relative abundance of cp 3–5 groups (K‐strategist nematodes) was highest under moderate thinning, with both groups at intermediate levels under heavy thinning. Characterized by rapid reproduction and short generation cycles, r‐strategist nematodes can quickly adapt to the nutrient pulse effects induced by light thinning (Bongers 1999). Under moderate thinning, shifts in the soil environment favor K‐strategist nematodes over r‐strategist nematodes (Pan et al. 2025), leading to a decrease in the relative abundance of cp 1–2 groups. This directional shift along the thinning intensity gradient aligns with the results of Principal Coordinates Analysis (PCoA), where the control was distinctly separated from the thinning treatments, while light, moderate, and heavy thinning samples exhibited a continuous distribution along the first principal coordinate, transitioning from r‐strategist dominance to K‐strategist dominance, reflecting that their community structures represent a gradient transitioning from r‐strategist to K‐strategist dominance. This confirms that the gradient of thinning intensity drives the directional shift in nematode life history strategies by modulating soil environmental stability.

## 5 Conclusion

This study systematically analyzed the differences in soil nematode community structure and their regulatory mechanisms under varying long‐term thinning intensities in a *Pinus taiwanensis* plantation. The results demonstrated that moderate thinning was the most effective in enhancing soil nematode community diversity and stability, thereby providing robust validation for the “intermediate disturbance hypothesis.” Under this treatment, the trophic structure of the nematode community was more intricate, and the functional state of the soil food web was more stable—primarily driven by the directional regulation of soil resource patterns. Although soil total phosphorus content was relatively lower under moderate thinning, the higher total organic carbon content provided a resource base for fungivorous nematodes and higher trophic level omnivorous‐predatory nematodes, facilitating trophic cascades and enhancing food web complexity. Concurrently, this treatment induced a gradient shift in nematode life‐history strategies from r‐strategists to K‐strategists, further strengthening the community's self‐regulatory capacity. Moreover, an elevated nematode channel ratio reflected the synergistic enhancement of both bacterial and fungal decomposition pathways, which is conducive to soil organic matter turnover and energy flow within the ecosystem. The relative abundances of bacterivorous, fungivorous, and omnivorous‐predatory nematodes increased under moderate thinning. All soil nematode community indices exhibited nonlinear responses to varying thinning intensities, indicating that appropriate thinning can substantially improve soil nematode community structure and function. Collectively, these results demonstrate that moderate thinning effectively enhances soil biodiversity, food web complexity, and ecosystem stability by optimizing the soil environment and resource allocation. Our results should be interpreted with caution due to the constraints of single sampling and limited replicates. Further repeated sampling is required to validate the responses of nematode communities along the thinning intensity gradient.

## Author Contributions

**Ruohan Wang:** data curation (equal), visualization (equal), writing – original draft (equal). **Weiya Xue:** data curation (equal), investigation (equal), writing – original draft (equal). **Hui Zhou:** investigation (equal), visualization (equal). **Fanglong Su:** project administration (equal), validation (equal). **Cancan Zhao:** supervision (equal), writing – review and editing (equal). **Renhui Miao:** writing – review and editing (equal). **Lei Su:** funding acquisition (lead), methodology (lead).

## Funding

This work was supported by the Natural Science Foundation of Henan Province (No. 262300421736), Shennongjia Altitudinal Gradient Research Platform (No. KFJ‐SW‐YW028), and the Science and Technology Plan Joint Foundation of Henan (No. 252301420029).

## Conflicts of Interest

The authors declare no conflicts of interest.

## Data Availability

The data of this study are available in Figshare https://doi.org/10.6084/m9.figshare.32657715.
